# Prenatal Exposure to Environmental Tobacco Smoke and Early Development of Children in Rural Guizhou Province, China

**DOI:** 10.3390/ijerph15122866

**Published:** 2018-12-14

**Authors:** Yang He, Renfu Luo, Tianyi Wang, Jingjing Gao, Chengfang Liu

**Affiliations:** 1 School of Advanced Agricultural Sciences, Peking University, Beijing 100871, China; heyangivy@pku.edu.cn (Y.H.); tianyi_34@163.com (T.W.); gaojj@pku.edu.cn (J.G.); cfliu.ccap@pku.edu.cn (C.L.); 2 The college of Economics and Business administration, Jiangxi Agricultural University, Nanchang 330029, China

**Keywords:** prenatal exposure to ETS, environmental tobacco smoke, early development

## Abstract

Background: There is a substantial body of evidence supporting the association between maternal active smoking during pregnancy and child development, but the association between prenatal exposure to environmental tobacco smoke (ETS) and early child development has not been well documented. This cross-sectional study examines the association between prenatal exposure to ETS and the development of children in their first two years of life. Methods: We interviewed the primary caregivers of 446 children under two years old in rural Guizhou Province, China. Based on self-reported assessments about whether the mother was exposed to ETS during pregnancy, we divided the children into the ETS-exposed group or the non-exposed group. Sociodemographic information was collected through a questionnaire. The cognitive, language, motor, and socioemotional abilities of children were assessed using the Bayley Scales of Infant Development III (BSID-III). A multivariate linear regression model adjusting for confounding variables was used to estimate the association of interest. Results: About 60% of mothers experienced ETS exposure during pregnancy. Cognitive and language scores were lower among children in the ETS-exposed group. When adjusting for characteristics of the child, the mother, the household, and village fixed effects, prenatal exposure to ETS was associated with lower cognition scores (−3.41; 95% confidence interval (CI): −6.39 to −0.42; *p* = 0.03) and language scores (−3.01; 95% CI: −5.39 to −0.09; *p* = 0.04). Frequency of prenatal exposure to ETS was also negatively associated with language development (−0.48; 95% CI: −0.87 to −0.09; *p* = 0.02) before children reached two years old. Conclusions: Prenatal exposure to ETS is negatively associated with the cognitive and language development of rural young children within their first two years of life. The government should take action to raise public awareness about the negative effects of tobacco use, with an emphasis on the protection of pregnant women and their children, in order to carry through comprehensive smoke-free laws in rural areas, while also increasing tobacco taxation.

## 1. Introduction

The high prevalence of tobacco use, including active smoking and environmental tobacco smoke (ETS) exposure, remains one of the leading risk factors for global mortality. In 2015, 25.0% of men and 5.4% of women worldwide smoked every day [1]. It is estimated that 40% of children, 33% of male nonsmokers, and 35% of female nonsmokers worldwide were exposed to ETS in 2004 [2]. In China, the prevalence of tobacco use is even higher: more than 300 million smokers (52.9% of men and 2.4% of women) and 740 million nonsmokers (55.19%) were exposed to ETS in 2010 [3,4].

Tobacco use during pregnancy has been found to affect development during infancy and early childhood. Development in utero and the first two years of life represents the most sensitive and vulnerable period for human growth and genetic–environmental interactions [5,6,7]. As a result, the first 1000 days of life may be more susceptible to the negative effects of thousands of harmful environmental toxicants found in tobacco, including lead, acetone, arsenic, tar, carbon monoxide, and nicotine in cigarette smoke [8,9,10].

Recently, concerns about the adverse effects of tobacco use during pregnancy on early childhood development have been growing in academia [11,12,13]. Many studies have shown a negative association between maternal active smoking during pregnancy and the development of children [11,14,15,16]. More specifically, there is a well-documented negative association between active smoking and cognitive verbal skills and language development [17,18,19,20].

While the impact of active maternal smoking on child development is well established, the association between prenatal ETS exposure and child development remains mixed [21]. Some studies show that prenatal exposure to ETS has been associated with negative childhood psychosocial development, such as in learning and memory [22]; delinquent, aggressive, and externalizing behaviors [23]; poor preschool temperament and erratic emotionality [24]; negative cognitive, language, fine-motor, and social–emotion development [25,26,27]; and low mental development index scores [28,29]. Other research has found no association after adjusting for the mother’s age, educational attainment, and annual family income. Still, other research has found a negative association among older children (seven years old), but not younger ones (four years old) [30,31,32].

In the context of China, although a couple of studies have been conducted in rural areas, the current evidence base is limited and mixed. Of the two studies presenting an association between prenatal exposure to ETS and the early development of children, one reported a negative association, while the other reported no association [30,31,32,33]. Some studies only assessed the effect of prenatal exposure to ETS on infants’ physiological index or on pregnancy outcomes [34,35]. In this study, we use the Bayley Scales of Infant Development III (BSID-III) to assess the developmental status of 446 sampled children younger than two years of age. We aimed to address the knowledge gap by adding more evidence on the association between prenatal exposure of ETS and early development of children under two years old from rural areas in southwestern China.

## 2. Materials and Methods

### 2.1. Study Participants

**Figure 1:**
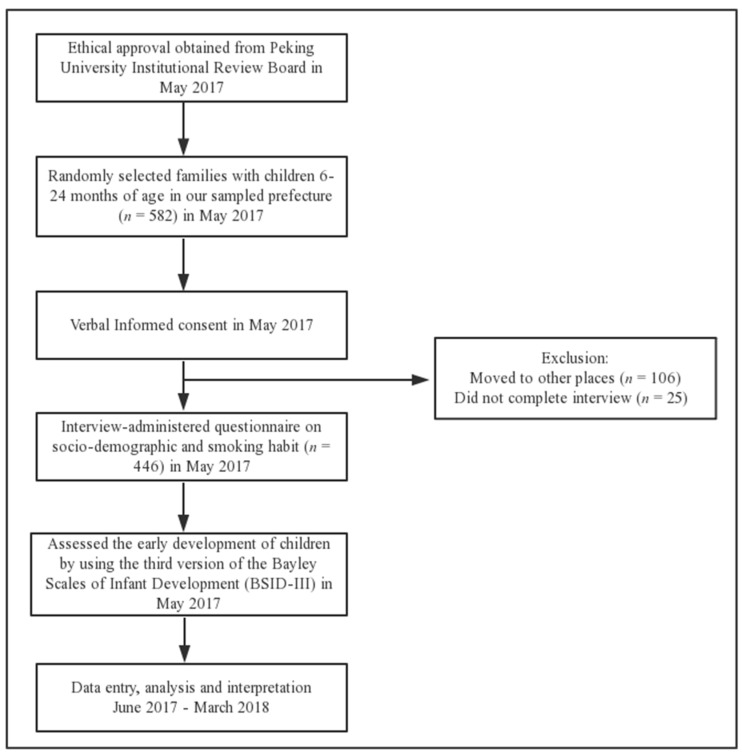
Flowchart of this study.

The current cross-sectional study was conducted in 41 rural settlements in nine villages of a township in the northwest part of Guizhou Province in southwest China in 2017. The sampled township is located in a prefecture with an average level of per capita income [36]. We obtained a list of 582 children who were in our desired age range from the county health and family planning commission. During home visits, we found that out of the 582 children, 111 had migrated to other places with their parents, and 25 did not complete the interview, as they were out of town to see doctors or visit relatives. We assessed 446 children and interviewed their mothers or primary caregivers (Figure 1).

The Peking University Institutional Review Board (PU IRB), Beijing, China, approved the ethical assessment of the study (No. IRB00001052-17056), and verbal informed consent was obtained from all of the study subjects.

### 2.2. Data Collection

The survey team collected three types of information: data from a socioeconomic survey; scores on cognitive, language, motor, and socioemotional development; and prenatal ETS exposure. Trained and assessed enumerators were sent to each sampled household to conduct the survey with the child’s primary caregiver; that is, the person who is most responsible for the child’s daily life. Enumerators were not permitted to do their work until strict training, assessments, field simulations, and pilot surveys were completed in order to ensure uniform manner and attitude during interviews in different households. The research protocols and questionnaire were developed and modified by the research team based on years of experience in field research. Quality control approaches such as carefully designed logic jumps and a triple-check system (self-check–intragroup check–team leader check) were implemented during and after field work.

#### 2.2.1. Socioeconomic Survey and Prenatal Exposure to ETS

The socioeconomic survey collected detailed information not only on the basic demographic characteristics and socioeconomic status of the children and their mothers, but also on household conditions. We also asked whether or not the mothers ever smoked or were exposed to ETS throughout the pregnancy, and the frequency of prenatal ETS exposure, measured by packs of cigarettes smoked by other family members per week during the pregnancy. Based on this self-reported information about prenatal ETS exposure, the children were divided into two groups: the ETS-exposed group and the non-exposed group.

#### 2.2.2. Outcome Measures

The children’s development was assessed based on the Bayley Scales of Infant Development III (BSID-III), which is an elaborate and comprehensively examined scale with sufficient reliability and validity. It is often considered the gold standard when assessing the development of children any time before 42 months of age [37]. The BSID-III results are categorized into four standardized scales: cognitive, language, motor, and socioemotional. The scales are age-standardized. The cognitive scale assesses play skills, information processing (attention to novelty, habituation, memory, and problem-solving), and counting and number skills [38]. The language scale assesses communication skills, including language and gestures [38]. The motor scale assesses fine and gross motor skills [38]. The socioemotional scale assesses functional emotional skills such as self-regulation and the ability to use emotions in a purposeful manner [39]. The cognitive, language, and motor scales evaluate the child’s performance on a series of interactive tasks, whereas the socioemotional scale is based on the caregiver’s responses to a series of questions.

#### 2.2.3. Confounding Factors

Other factors may also lead to undesirable development outcomes in human infants, and women who were exposed to tobacco smoke during pregnancy are more likely to exhibit these risk factors, including less optimal socioeconomic status [24]. Previous studies have shown that adjusting for the characteristics of the child, the mother, and the household when examining the association between the status of prenatal exposure to ETS and early childhood development can help increase the efficiency of estimation [40,41]. Following the above literature, we introduced three categories of variables as confounding factors in the multivariate linear regression: (1) child characteristics: gender (boy/girl), age in months (mean ± standard deviation (SD)), and low birth weight (yes/no); (2) mother characteristics: age in years and education attainment (greater than nine years/at most nine years); and (3) household characteristics: mother is the primary caregiver (yes/no), and family income in the last year (greater than 25,000 yuan/at most 25,000 yuan). In this study, low birth weight is defined as less than 2500 g [42].

### 2.3. Statistical Analysis

Continuous variables were presented as mean ± standard deviation as compared by Student’s t-test. Categorical variables were presented as numbers (percent) and compared among prenatally ETS-exposed and non-exposed groups using the Chi-square test. We performed multivariate linear regression, with cognitive, language, and motor development scores as dependent variables in separate analyses, and self-reported prenatal exposure to ETS as an independent variable. Statistical analyses were performed using Stata 14.2 software. The significance level was set to *p* < 0.05 (two-tailed).

The unadjusted linear regression model is as follows:

(1) $$Y_{i}=\beta_{0}+\beta_{1}ETS_{i}+\epsilon_{i}$$

where $Y_{i}$ denotes the development of child *i*, including BSID-III standardized scores on cognition, language, and motor and socioemotional developments. $ETS_{i}$ is the self-reported prenatal ETS exposure of the mother, and $\epsilon_{i}$ is the error term of the model, which is clustered at the natural village level and adjusted for village fixed effect.

The adjusted linear regression model is as follows:

(2) $$Y_{i}=\beta_{0}+\beta_{1}ETS_{i}+\beta_{2}CHILD_{i}+\beta_{3}MOTHER_{i}+\beta_{4}HOUSEHOLD_{i}+\epsilon_{i}$$

where $CHILD_{i}$ is a vector of child characteristics, including gender, age in months, and low birth weight (yes/no); $MOTHER_{i}$ is a vector of maternal characteristics, including age and level of education; and $HOUSEHOLD_{i}$ is a vector of household characteristics, including whether the mother is the primary caregiver (yes/no), and the family income in the last year. The meanings of $Y_{i}$*,* $ETS_{i}$, and $\epsilon_{i}$ are the same as those in Equation (1).

The hypothesis drawn from Equation (2) is that $\beta_{1}$ does not equal zero, indicating that early childhood development is associated with prenatal exposure to ETS. The variance inflation factor (VIF) was examined to test multicollinearity. The results are presented in Table A1. We can see that the VIF values are all less than two, which is far less than the critical value of 10 [43]. These results indicate that there is no multicollinearity in the multivariate linear regression model.

## 3. Results

According to the self-reported questionnaire, we found that none of the sampled mothers identified themselves as active smokers during pregnancy. Of 446 child–caregiver dyads included in our study, 270 children (60.5%) were born to mothers exposed to ETS during pregnancy (Table 1). There is no significant difference in the children’s gender, age, and birth weight between the ETS-exposed group and non-exposed group. Similarly, no significant association could be detected between self-reported prenatal exposure to ETS and maternal education, mother as the primary caregiver, and family income in the last year.

**Figure A1:**
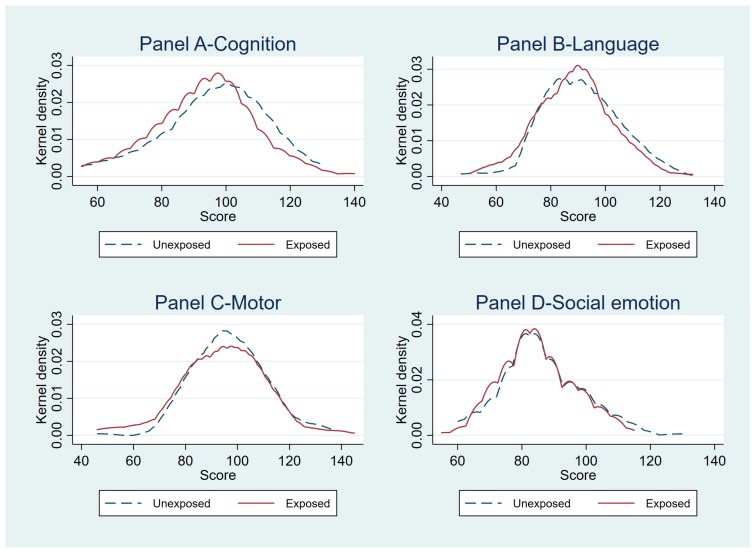
The distribution of the Bayley Scales of Infant Development III (BSID-III) test score among children with and without ETS (environmental tobacco smoke) exposure during pregnancy.

**Figure A2:**
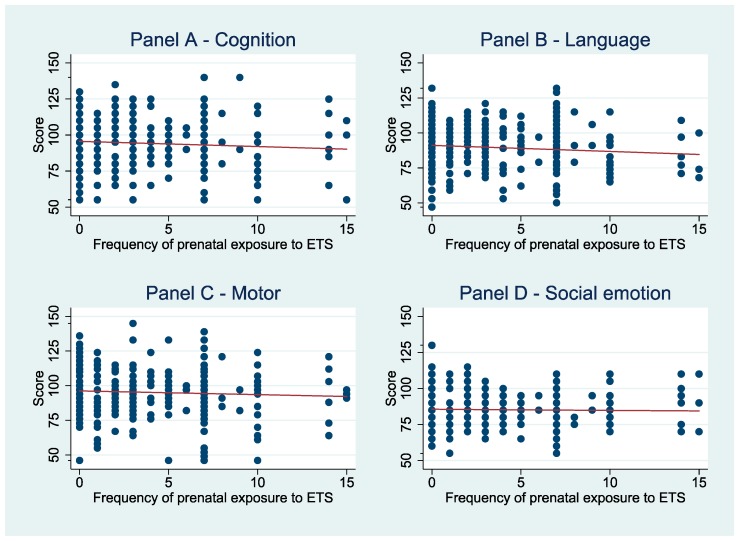
The association between BSID-III test score and the frequency of exposure to ETS during pregnancy.

The density distribution curves of the four BSID-III standardized scales (Figure A1) gave us a clear sense of a systematic relationship between prenatal ETS exposure and the early cognitive and language development of children, while the frequency of prenatal exposure to ETS seems to be negatively associated with children’s language and cognition scores (Figure A2).

Our data show that compared with their younger cohorts, children older than 15 months revealed lower cognition scores (91.5; 95% CI: 75.8–107.2; *p* < 0.01 vs. 97.3; 95% CI: 80.9–113.7; *p* < 0.01; Table 2) and language scores (88.2; 95% CI: 76.2–100.2; *p* = 0.02 vs. 91.2; 95% CI: 76.7–105.7; *p* = 0.02). However, infants older than 15 months had higher motor scores (100.5; 95% CI: 86.7–114.3; *p* < 0.01 vs. 90.4; 95% CI: 74.4–106.4; *p* < 0.01). Compared with children whose mothers were not the primary caregivers, children whose mothers were the primary caregivers had higher cognitive scores (96.1; 95% CI: 79.2–113; *p* = 0.04 vs. 92.9; 95% CI: 77.5–108.3; *p* = 0.04) and language scores (91.9; 95% CI: 77.4–106.4; *p* < 0.01 vs. 87.7; 95% CI: 75.2–100.2; *p* < 0.01).

The multivariate linear regression for the effect of prenatal exposure to ETS on child development (Table 3) shows that children born to mothers exposed to ETS experienced worse cognitive development (−3.41; 95% CI: −6.39 to −0.42; *p* = 0.03) and language development (−3.01; 95% CI: −5.93 to −0.09; *p* = 0.04) than those whose mothers were not exposed to ETS during pregnancy. The frequency of prenatal exposure to ETS had a significant negative association with children’s early language development (−0.48; 95% CI: −0.87 to −0.09; *p* = 0.02; Table 4). That is, for each additional pack of cigarettes smoked per week by family members in the household, the child’s language score potentially drops by 0.48 points in early childhood, after controlling for confounding variables. Based on the robustness check (As a robustness check, we conducted multivariate analyses using five empirical specifications. In the first empirical specification of Table A2, no control variables were introduced. In the second empirical specifications of the table, the characteristics of child, mother, and household were controlled for simultaneously. In Table A3, there are three empirical specifications, which means the child’s, mother’s, and household’s characteristics were controlled for, one at a time. Table A2 and Table A3 show that the results from analyzing the associations between prenatal ETS exposure and early childhood development are robust to model specifications. Therefore, as we did in the original, we reported results from these two empirical specifications in Table A2), we can see that the association between prenatal exposure to ETS and early childhood development remains substantially unchanged.

## 4. Discussion

Our study found that none of the mothers surveyed reported themselves to be active smokers. Our study also found that 60.5% of mothers of children younger than two years in rural Guizhou reported prenatal exposure to ETS, which is higher than the national average of 52% for all adults found by the International Tobacco Control China Survey [44]. Nevertheless, according to the China Report on the Health Hazards of Smoking Executive Summary, two-thirds of the Chinese population are unaware of the threat of ETS [45]. Exposure to environmental tobacco smoke is indeed a matter of concern for researchers and policy makers.

When comparing the two groups of children by socioeconomic status, we found that children older than 15 months had significantly higher motor scores and lower cognitive and language scores than those under 15 months. Insufficient parenting might have played some role in this phenomenon. Similar results were also found in the literature. For example, a study found that in Shaanxi Province, caregivers often placed greater importance on feeding and clothing their children instead of playing and talking with them [46].

Consistent with earlier studies, our data provide evidence to support the argument that prenatal exposure to ETS is negatively correlated with children’s cognitive development [10,25,26,27,28,30]. Our study also reached the same conclusion as earlier studies that children’s language abilities were related to prenatal exposure to ETS [10,25,27].

However, we did not find any association between prenatal exposure to ETS and children’s motor development. This result is consistent with Christensen et al. [47], who assessed prenatal smoking exposure via the maternal serum cotinine, and found that delayed motor function abilities in children before school age was not related to prenatal exposure to ETS. Nonetheless, findings from the Polish Mother and Child Cohort study suggest that children’s motor abilities by 12 and 24 months decrease significantly in relation to ETS exposure [48]. Differences in those results could potentially be attributed to various exposure and outcome assessments, the child’s age, or the selection of confounding variables. Thus, more studies are needed in order to investigate the underlying biological and psychopathological mechanisms, with careful and well-defined research design.

We found that the self-reported frequency of prenatal exposure to ETS is important when analyzing its association with child language development. Similar results were reported by Jedrychowski et al., who measured smoking frequency by the number of cigarettes smoked per day by other household members at home over the duration of the pregnancy [28]. Using data from the Collaborative Perinatal Project, with 52,919 children aged 4 and 7, Gilman and colleagues reported that after distinguishing by exposure intensity (0, one to nine, 10–19, or ≥20 cigarettes per day), heavy exposure (defined as ≥20 cigarettes per day) was associated with a relatively lower intelligence score for four-year-old children [30].

The mechanisms linking the association between prenatal exposure to ETS and early childhood development are likely complex. The biomedical literature has proposed at least two likely mechanisms. One is that in addition to the well-known presence of nicotine, tobacco contains many other toxic substances, such as hydrocyanic acid, ammonia, carbon monoxide, carbon dioxide, pyridine, and so on [49,50]. These toxic substances are absorbed into the mother’s blood with the smoke, increasing her carboxyhemoglobin levels and reducing the uteroplacental blood flow and oxygen-carrying capacity, which may lead to fetal hypoxia [51]. The other mechanism is that the toxic chemicals in ETS can affect the developing nervous system [52]. This damage might be one of the risk factors of later cognitive and language developmental delay during early childhood.

This study makes additional contributions to the evidence base on the adverse neurocognitive association of prenatal exposure to ETS in rural Guizhou Province, where the smoking rate remains quite high, but public awareness is lagging behind. To our knowledge, this study is the first to explore the association between prenatal exposure to ETS and early childhood development in rural Guizhou, which is a relatively distant and economically less developed area in China. Translation, back translation, and cultural adaptation were done to the Chinese version of the harmonized Bayley Scales of Infant Development III to ensure that the four scales of early childhood development would not be overestimated or underestimated under local circumstances.

We acknowledge certain limitations of our study. Prenatal exposure to ETS was measured based on maternal self-reported information. Our data do not contain any detailed information on the intensity or frequency of children’s direct exposure to ETS after the mother gives birth, the distance between pregnant women and their smoking family members, or whether women were exposed to ETS outside the home, in particular in the workplace. It is likely that family members who smoke during the pregnancy also tend to smoke after the child is born. A report shows that the quitting rate of smoking is 7.9% among male smokers in Guizhou Province [36]. According to the International Tobacco Control China Survey, 75.6% of Chinese smokers do not intend to quit [53]. Given the nature of the cross-sectional design and the self-reported measurement of prenatal exposure to ETS, we were not able to identify whether there is any causal relationship between prenatal exposure to ETS and the outcome variables of interest. Biomarkers such as the nicotine or cotinine content in maternal blood or hair are more reliable to measure such exposure.

Earlier studies have pointed out several possible tobacco control policies. One study indicated that the tax rate of tobacco as a percentage of the retail price is still relatively low compared to the tax rate recommended by the World Health Organization [54], which means there is still some room left for the government to further increase tobacco taxation so as to reduce the number of smokers in China. A meta-analysis reported that the implementation of comprehensive smoke-free laws was associated with reductions in the rates of several childhood infections [55]. Therefore, enacting comprehensive smoke-free laws might be a better policy choice. Due to a large portion of Chinese people not realizing the harm of tobacco use [44], the government might also consider interventions to raise public awareness about the health burden of ETS exposure, such as graphical warnings and mass media campaigns. From this study, we found that in rural Guizhou, the rates of smoking and ETS exposure are relatively high, whereas the regulation of smoking in public places is insufficiently practiced. The government should take action to raise public awareness about the negative effects of tobacco use, with an emphasis on the protection of pregnant women and their children, and enact comprehensive smoke-free laws in rural areas, while increasing tobacco taxation.

## 5. Conclusions

In conclusion, we evaluated prenatal exposure to ETS using a self-reported survey with mothers in rural China, and found a negative association with children’s cognitive and language development before the age of two. The frequency of prenatal exposure to ETS is also negatively correlated with children’s language development. Such results suggest that the government should consider pregnant women as the focus of attention when launching environmental tobacco smoke interventions, especially in rural areas.

## Figures and Tables

**Table 1:** Comparison of socioeconomic characteristics between children with and without maternal prenatal exposure to ETS (environmental tobacco smoke).

Variables		Prenatal Exposure to ETS?		*p*-Value
		No (*n* = 176)	Yes (*n* = 270)	
Sex	Boy	105 (59.7)	152 (56.3)	0.48
	Girl	71 (40.3)	118 (43.7)	
Age of child in month (Mean ± SD)		14.8 ± 5.6	14.5 ± 5.4	0.59
Low birth weight	No	160 (91.9)	254 (94.1)	0.39
	Yes	14 (8.1)	16 (5.9)	
Age of mother in year (Mean ± SD)		24.7 ± 5.6	24.5 ± 4.9	0.60
Education of mother	≤9 years	151 (85.8)	242 (89.6)	0.22
	>9 years	25 (14.2)	28 (10.4)	
Is mother primary caregiver	No	90 (51.7)	132 (48.9)	0.56
	Yes	84 (48.3)	138 (51.1)	
Family income in the last year (RMB)	≤25,000 Yuan	71 (40.6)	107 (39.6)	0.84
	>25,000 Yuan	104 (59.4)	163 (60.4)	

SD, Standard deviation; numbers in parenthesis are percentages for categorical variables.

**Table 2:** Comparison of child development by socioeconomic characteristics of sampled children.

Variables		Cognition Score		Language Score		Motor Score		Social Emotional Score	
		(Mean ± SD)	*p*-Value	(Mean ± SD)	*p*-Value	(Mean ± SD)	*p*-Value	(Mean ± SD)	*p*-Value
Sex	Boy	95.6 ± 16.5	0.08	88.9 ± 13.7	0.17	96.2 ± 15.4	0.19	85.3 ± 12.3	0.95
	Girl	92.9 ± 16.0		90.7 ± 14.0		94.2 ± 16.2		85.4 ± 11.4	
Age of child	≤15 months	97.3 ± 16.4	<0.01	91.2 ± 14.5	0.02	90.4 ± 16.0	<0.01	84.4 ± 12.2	0.10
	>15 months	91.5 ± 15.7		88.2 ± 12.0		100.5 ± 13.8		86.3 ± 11.5	
Low birth weight	No	94.9 ± 16.0	0.05	90.0 ± 13.5	0.13	95.7 ± 15.5	0.14	85.7 ± 11.7	0.07
	Yes	89.0 ± 18.0		86.1 ± 16.5		91.3 ± 18.6		81.7 ± 13.7	
Age of mother	≤25 years	95.2 ± 15.7	0.23	89.9 ± 13.4	0.65	95.4 ± 15.8	0.90	85.7 ± 11.9	0.33
	>25 years	93.2 ± 17.3		89.3 ± 14.6		95.2 ± 15.8		84.5 ± 11.9	
Education of mother	≤9 years	93.9 ± 16.5	0.04	89.4 ± 13.7	0.23	95.1 ± 15.9	0.33	85.1 ± 12.1	0.19
	>9 years	98.8 ± 14.6		91.8 ± 14.5		97.3 ± 14.7		87.4 ± 10.4	
Is mother primary caregiver	No	92.9 ± 15.4	0.04	87.7 ± 12.5	<0.01	96.1 ± 15.2	0.38	86.0 ± 12.4	0.32
	Yes	96.1 ± 16.9		91.9 ± 14.5		94.8 ± 16.3		84.8 ± 11.3	
Family income in the last year (RMB)	≤ 25,000 Yuan	91.7 ± 14.7	<0.01	88.4 ± 13.6	0.11	93.9 ± 15.8	0.11	85.6 ± 12.4	0.70
	>25,000 Yuan	96.3 ± 15.8		90.5 ± 13.9		96.3 ± 15.7		85.2 ± 11.6	

SD, Standard deviation; numbers in parenthesis are percentages for categorical variables.

**Table 3:** Multivariate regression for prenatal exposure to ETS on child development in rural Guizhou province.

Dependent Variables	Unadjusted Analysis (ETS = 1) ^a^		Adjusted Analysis (ETS = 1) ^a,b^	
	Coefficient (95% CI)	*p*-Value	Coefficient (95% CI)	*p*-Value
Cognition score	−2.75 (−5.40, −0.11)	0.04	−3.41 (−6.39, −0.42)	0.03
Language score	−2.62 (−5.36, 0.13)	0.06	−3.01 (−5.93, −0.09)	0.04
Motor score	−2.78 (−5.74, 0.71)	0.06	−2.52 (−5.43, 0.38)	0.09
Social emotion score	−0.75 (−3.02, 1.52)	0.51	−0.88 (−3.29, 1.53)	0.46

^a^ Standard errors are clustered at the natural village level and adjusted for village fixed effect. ^b^ Regression estimates from multivariate linear models adjusted for gender, age, birth weight, age and education of mother, whether mother is primary caregiver, family income, and village fixed effect.

**Table 4:** Multivariate regression for the self-reported frequency of prenatal exposure to ETS on child development in rural Guizhou province.

Dependent Variables	Unadjusted Analysis (Packs of Cigarettes per Week) ^a^		Adjusted Analysis (Packs of Cigarettes per Week) ^a,b^	
	Coefficient (95% CI)	*p*-Value	Coefficient (95% CI)	*p*-Value
Cognition score	−0.33 (−0.80, 0.14)	0.17	−0.33 (−0.76, 0.11)	0.14
Language score	−0.45 (−0.82, −0.08)	0.02	−0.48 (−0.87, −0.09)	0.02
Motor score	−0.27 (−0.71, 0.17)	0.22	−0.31 (−0.70, 0.08)	0.12
Social emotion score	0.002 (−0.32, 0.33)	0.99	−0.002 (−0.36, 0.36)	0.99

^a^ Standard errors are clustered at the natural village level and adjusted for village fixed effect. ^b^ Regression estimates from multivariate linear models adjusted for gender, age, birth weight, age, and education of mother, whether mother is primary caregiver, family income, and village fixed effect.

## Appendix A

**Table A1:** The VIF values of the multicollinearity test of linear regression model with confounding variables.

Variables	VIF Values
ETS/ Packs of cigarettes per week	1.06
Child’s gender (male = 1)	1.10
Age of child in months	1.57
Low birth weight (yes = 1)	1.08
Mother’s age in years	1.15
Education attainment of the mother	1.09
If mother is the primary caregiver of the child	1.19
Family income in the last year	1.12
Mean VIF	1.50

Notes: VIF, variance inflation factor.

**Table A2:** Results of unadjusted and adjusted regressions on early development of children in rural Guizhou province.

Independent Variables	Model 1		Model 2	
	Coefficient (95% CI)	*p*-Value	Coefficient (95% CI)	*p*-Value
Panel A: Cognition score				
Prenatal exposure to ETS (Yes = 1; No = 0)	−2.75 (−5.40, −0.11)	0.04	−3.41 (−6.39, −0.42)	0.03
Child characteristics	N		Y	
Mother characteristics	N		Y	
Household characteristics	N		Y	
Constant	Y		Y	
Frequency of prenatal exposure to ETS	−0.33 (−0.80, 0.14)	0.17	0.33 (−0.76, 0.11)	0.14
Child characteristics	N		Y	
Mother characteristics	N		Y	
Household characteristics	N		Y	
Constant	Y		Y	
Panel B: Language score				
Prenatal exposure to ETS (Yes = 1; No = 0)	−2.62 (−5.36, 0.13)	0.06	−3.01 (−5.93, −0.09)	0.04
Child characteristics	N		Y	
Mother characteristics	N		Y	
Household characteristics	N		Y	
Constant	Y		Y	
Frequency of prenatal exposure to ETS	−0.45 (−0.82, −0.08)	0.02	−0.48 (−0.87, −0.09)	0.02
Child characteristics	N		Y	
Mother characteristics	N		Y	
Household characteristics	N		Y	
Constant	Y		Y	
Panel C: Motor score				
Prenatal exposure to ETS (Yes = 1; No = 0)	−2.78 (−5.74, 0.71)	0.06	−2.52 (−5.43, 0.38)	0.09
Child characteristics	N		Y	
Mother characteristics	N		Y	
Household characteristics	N		Y	
Constant	Y		Y	
Frequency of prenatal exposure to ETS	−0.27 (−0.71, 0.17)	0.22	−0.31 (−0.70, 0.08)	0.12
Child characteristics	N		Y	
Mother characteristics	N		Y	
Household characteristics	N		Y	
Constant	Y		Y	
Panel D: Social emotion score				
Prenatal exposure to ETS (Yes = 1; No = 0)	−0.75 (−3.02, 1.52)	0.51	−0.88 (−3.29, 1.53)	0.46
Child characteristics	N		Y	
Mother characteristics	N		Y	
Household characteristics	N		Y	
Constant	Y		Y	
Frequency of prenatal exposure to ETS	0.002 (−0.32, 0.33)	0.99	−0.002 (−0.36, 0.36)	0.99
Child characteristics	N		Y	
Mother characteristics	N		Y	
Household characteristics	N		Y	
Constant	Y		Y	

Data source: Authors’ survey; Notes: Y denotes that these confounding variables are included in the regression; N denotes that these confounding variables are not included in the regression. Child characteristics: gender, age in month, and low birth weight (yes/no); Mother characteristics: age, and education attainment; Household characteristics: mother is the primary caregiver (yes/no), and family income in the last year.

**Table A3:** Results of regressions with different confounding variables on early development of children in rural Guizhou province.

Independent Variables	Model 1		Model 2		Model 3	
	Coefficient (95% CI)	*p*-Value	Coefficient (95% CI)	*p*-Value	Coefficient (95% CI)	*p*-Value
Panel A: Cognition score						
Prenatal exposure to ETS (Yes = 1; No = 0)	−3.41 (−6.32, −0.50)	0.02	−2.71 (−5.47, 0.04)	0.05	−3.00 (−5.82, -0.18)	0.04
Child characteristics	Y		N		N	
Mother characteristics	N		Y		N	
Household characteristics	N		N		Y	
Constant	Y		Y		Y	
Frequency of prenatal exposure to ETS	−0.33 (−0.78, 0.12)	0.14	−0.32 (−0.78, 0.15)	0.17	−0.33 (−0.79, 0.12)	0.14
Child characteristics	Y		N		N	
Mother characteristics	N		Y		N	
Household characteristics	N		N		Y	
Constant	Y		Y		Y	
Panel B: Language score						
Prenatal exposure to ETS (Yes = 1; No = 0)	−2.97 (−5.82, −0.12)	0.04	−2.61 (−5.42, 0.12)	0.07	−2.89 (−5.74, −0.04)	0.05
Child characteristics	Y		N		N	
Mother characteristics	N		Y		N	
Household characteristics	N		N		Y	
Constant	Y		Y		Y	
Frequency of prenatal exposure to ETS	−0.48 (−0.86, −0.11)	0.01	−0.45 (−0.83, −0.07)	0.02	−0.46 (−0.83, −0.09)	0.02
Child characteristics	Y		N		N	
Mother characteristics	N		Y		N	
Household characteristics	N		N		Y	
Constant	Y		Y		Y	
Panel C: Motor score						
Prenatal exposure to ETS (Yes = 1; No = 0)	−0.32 (−0.70, 0.08)	0.11	−0.26 (−0.71, 0.18)	0.24	−0.28 (−0.72, 0.16)	0.20
Child characteristics	Y		N		N	
Mother characteristics	N		Y		N	
Household characteristics	N		N		Y	
Constant	Y		Y		Y	
Frequency of prenatal exposure to ETS	−0.32 (−0.70, 0.08)	0.11	−0.26 (−0.71, 0.18)	0.24	−0.28 (−0.72, 0.16)	0.20
Child characteristics	Y		N		N	
Mother characteristics	N		Y		N	
Household characteristics	N		N		Y	
Constant	Y		Y		Y	
Panel D: Social emotion score						
Prenatal exposure to ETS (Yes = 1; No = 0)	−0.87 (−3.28, 1.54)	0.47	−0.74 (−3.04, 1.56)	0.52	−0.91 (−3.18, 1.36)	0.43
Child characteristics	Y		N		N	
Mother characteristics	N		Y		N	
Household characteristics	N		N		Y	
Constant	Y		Y		Y	
Frequency of prenatal exposure to ETS	−0.0001 (−0.36, 0.36)	1.00	−0.006 (−0.31, 0.32)	0.97	−0.13 (−0.34, 0.32)	0.94
Child characteristics	Y		N		N	
Mother characteristics	N		Y		N	
Household characteristics	N		N		Y	
Constant	Y		Y		Y	

Data source: Authors’ survey; Notes: Y denotes that these confounding variables are included in the regression; N denotes that these confounding variables are not included in the regression. Child characteristics: gender, age in month, and low birth weight (yes/no); Mother characteristics: age, and education attainment; Household characteristics: mother is the primary caregiver (yes/no), and family income in the last year.
